# Supplementary material for: Semi-automated closed system manufacturing of lentivirus gene-modified haematopoietic stem cells for gene therapy
Source: Nat Commun. 2016 Oct 20;7:13173. doi: 10.1038/ncomms13173 (PMC5080442; doi:10.1038/ncomms13173)
Supplement: Supplementary Information — Supplementary Figures 1-5, Supplementary Tables 1-7 [file ncomms13173-s1.pdf]

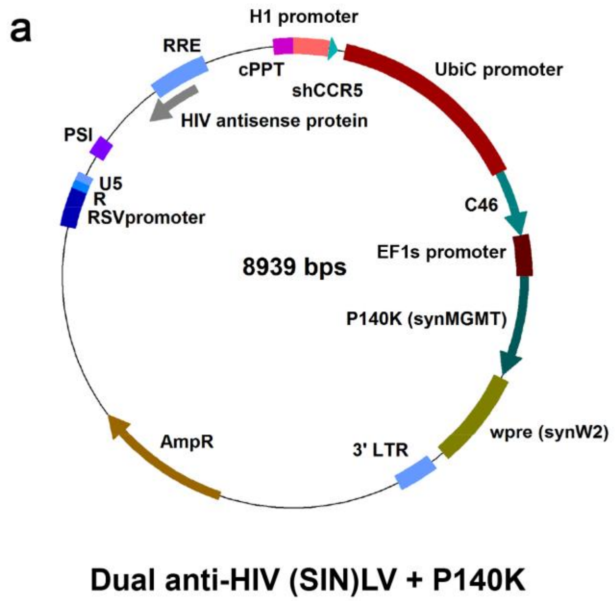

Supplementary Figure 1. Vector Maps. Both vectors are third-generation self-inactivating lentiviruses derived from the same HIV-1 backbone. (A) The clinical-grade LV encoding two anti-HIV transgenes (*shCCR5* and *C46*), as well as a synthetic O6-benzylguanine resistant *MGMT* mutant P140K transgene. (B) The *eGFP* and synthetic O_6-benzylguanine resistant *MGMT* mutant P140K transgene-encoding LV used for gene transfer and *in vivo* tracking in nonhuman primates. Both vectors lack the X' open reading frame present in the woodchuck hepatitis virus post-transcriptional response element (wpre). AmpR (ampicillin resistance gene); RSV (Rous Sarcoma Virus); PSI (encapsidation sequence); RRE (rev response element); cPPT (central polypurine tract); SFFV (Spleen Focus-forming Virus); PGK (phosphoglycerate kinase); LTR (long terminal repeat).

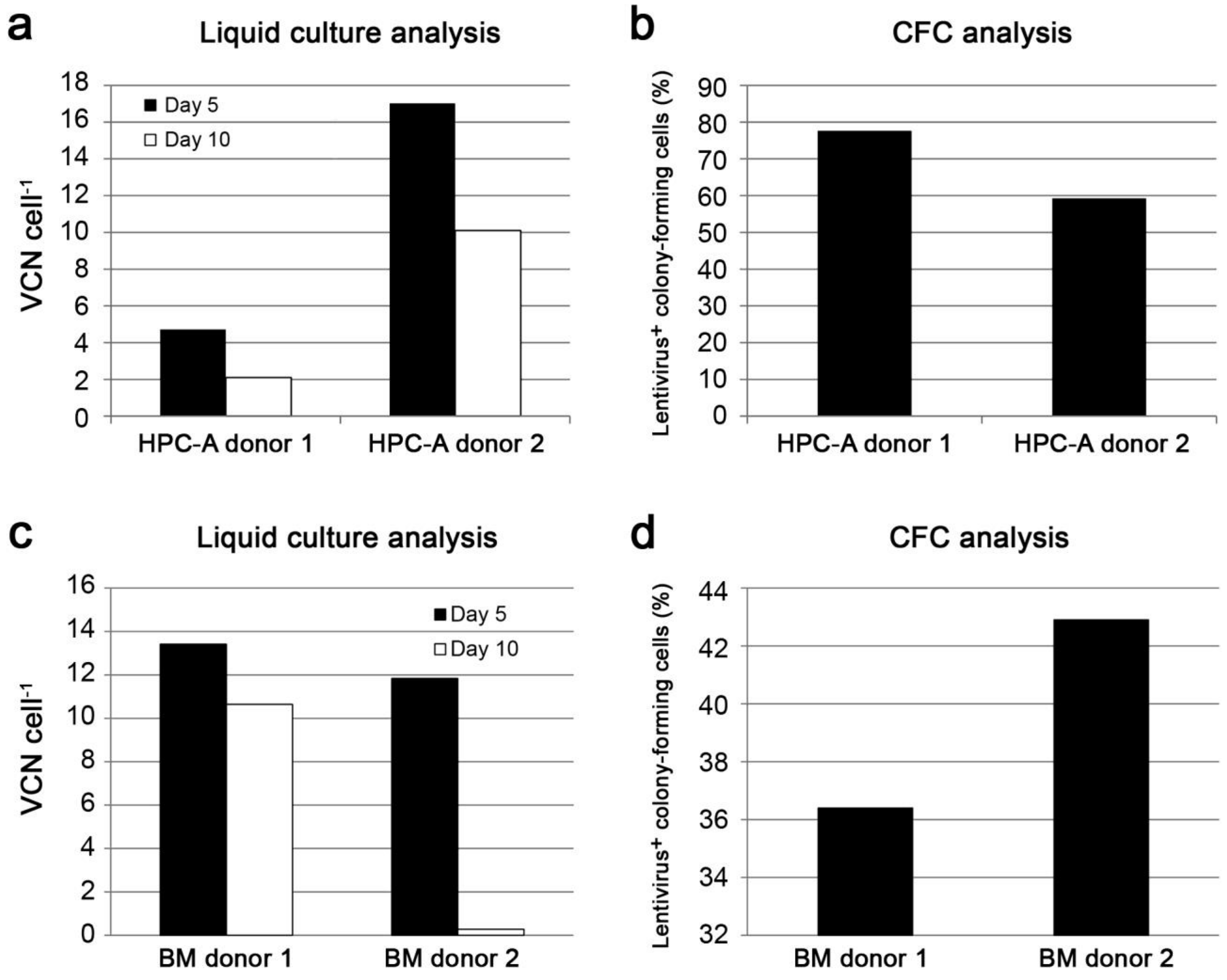

Supplementary Figure 2. Gene marking in CD34+ leukocytes following automated transduction. (**A** and **B**) Growth factor mobilized HPC-A products. (**C** and **D**) Bone marrow products. Following semi-automated transduction and harvest, aliquots of the final cell product were cultured in liquid media consisting of recombinant human growth factors GCSF, SCF, TPO, Flt3-L, IL-3 and IL-6 for subsequent real-time PCR to determine VCN (**A and C**), or in methylcellulose media containing the same recombinant human growth factors for colony forming assay (**B and D**) for subsequent real-time PCR to determine VCN.

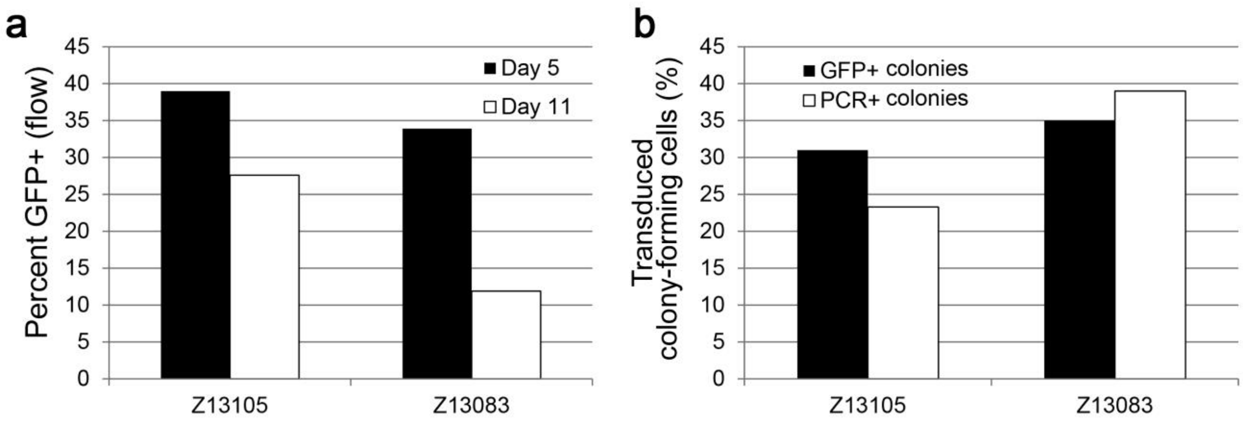

Supplementary Figure 3. Gene marking in growth factor primed bone marrow nonhuman primate CD34⁺ hematopoietic cells following automated transduction. Following semi-automated transduction and harvest, aliquots of the final cell product for infusion into myeloablated monkeys were cultured in liquid media consisting of recombinant human growth factors GCSF, SCF, TPO, Flt3-L, IL-3 and IL-6 for subsequent flow cytometry (**A**), or in methylcellulose media containing the same recombinant human growth factors for colony forming assay (**B**) and subsequent flow cytometry or PCR reactivity.

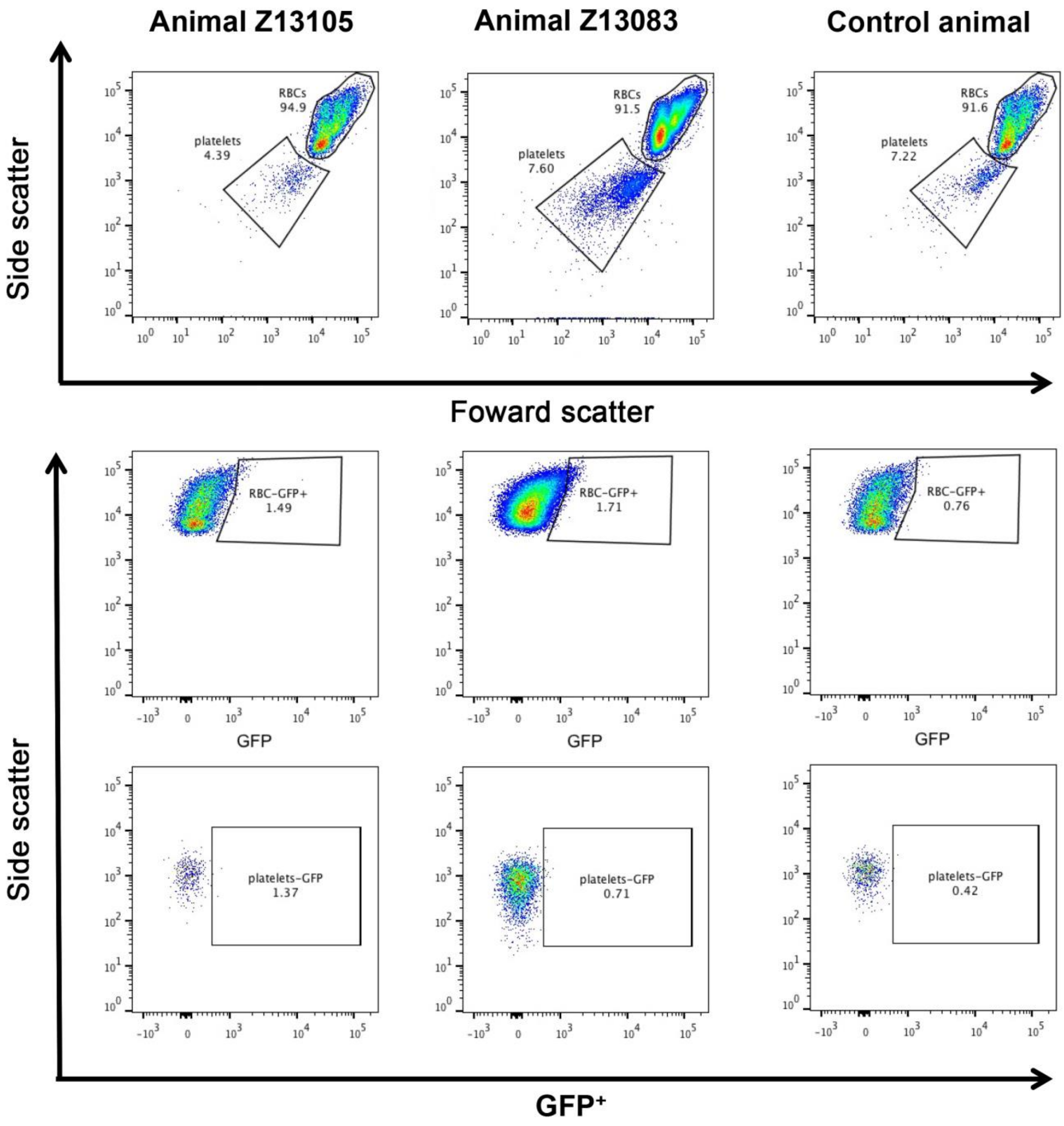

Supplementary Figure 4. Transgene expression in nonhuman primate red blood cells and platelets in vivo following automated transduction. At 258 and 222 days post-transplant for animals Z13105 and Z13083, respectively, flow cytometry revealed GFP transgene expression in RBC and platelets relative to a control (non-transplanted) animal (lower panel).

Upper panel represents scatter properties of RBC and platelets for each animal. Gates were established using the control animal.

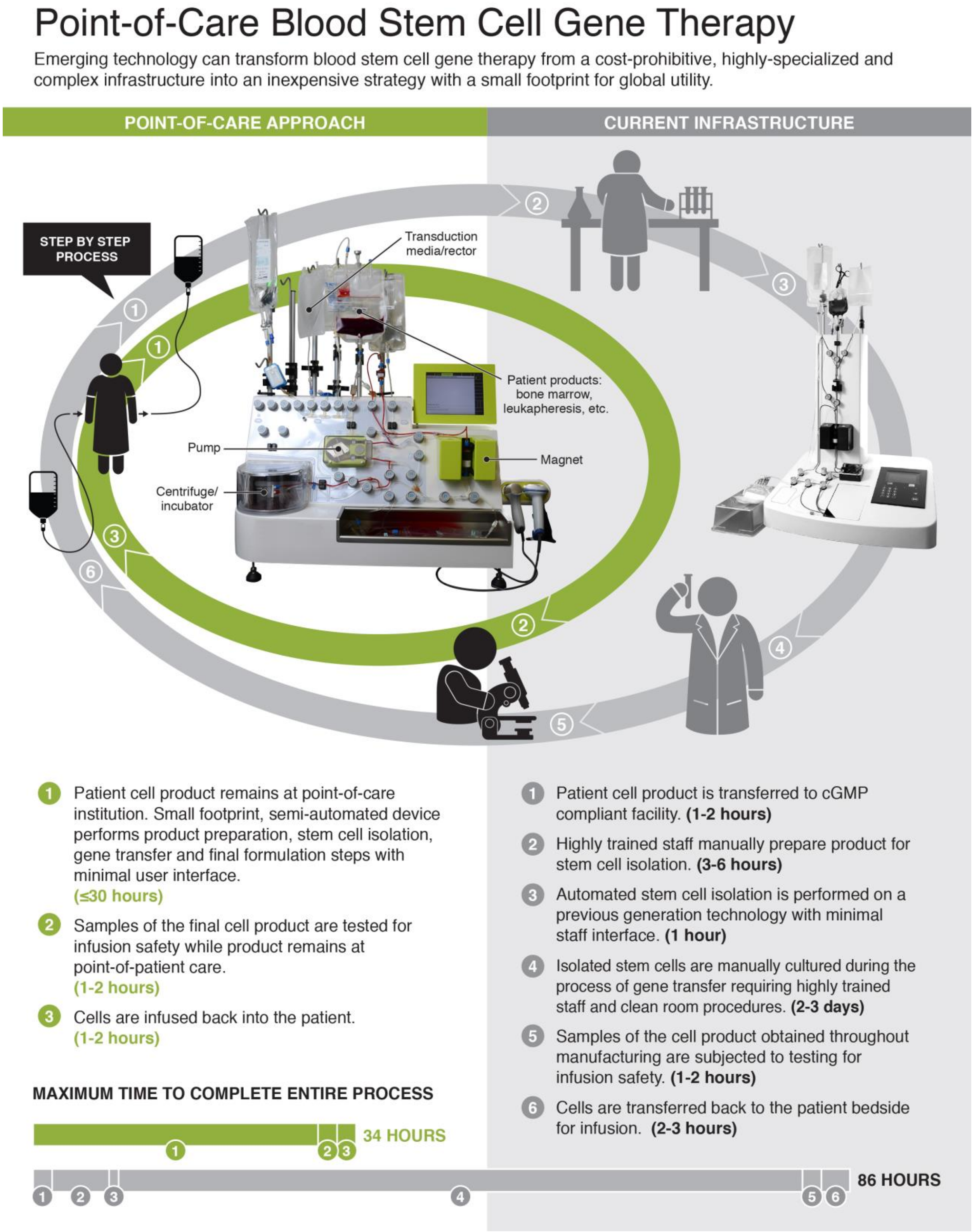

Supplementary Figure 5.

Supplementary Table 1: Complete Description of Custom, Flexible Program Inventory

Program Number	Program Description	Tubing Set
1	This program is suitable for RBC depletion of bone marrow or peripheral blood of initial volume ≥ 10mL. A HES red blood cell reduction methodology was chosen due to historical reporting of high CD34+ cell population recovery. Starting bone marrow/blood product at ≤ 25% HCT is mixed with 20% volume HES and PBS/EDTA buffer in stages depending on initial product volume. Each stage has a maximum volume of 300mL and includes a slow centrifugation step to initiate rouleaux, followed by slow transfer of product to funneled sedimentation bag provided by the user. Minimum sedimentation wait is 30 minutes and can be prematurely terminated or extended infinitely by the user through the touchscreen interface. Following desired sedimentation time, the user is prompted to initiate RBC-rich fraction removal in a stepwise fashion by user-defined volumes until the desired RBC pellet size is reached. Once the user confirms completion of RBC removal, the program automatically initiates supernatant washing in PBS/EDTA buffer to remove residual HES. Once washing is complete, the RBC depleted fraction is concentrated to the desired volume for bead labeling (~90mL) through a centrifugation and aspiration step and remains in the device chamber. We found that with this method the RBC pellet of even a large marrow product could be reduced to under 10ml.	TS100
2	This program is suitable for labeling of any desired cell fraction with a directly-conjugated immunomagnetic bead. The program initiates with the assumption that the cell product to be labeled is in the device chamber. The volume to be labeled is ~95mL. The cell fraction to be labeled is first diluted in PBS/EDTA buffer and then is pelleted in a step-wise fashion. Immunomagnetic beads (7.5mL) with or without blocking agent are then added and the chamber is cooled to 20°C and gently shaken every 30 seconds for a total incubation period of 30 minutes. Following incubation the labeled	TS100

Supplementary Table 1: Complete Description of Custom, Flexible Program Inventory

Program Number	Program Description	Tubing Set
	cell fraction is centrifuged and washed to remove non-bound excess beads. Once washing is complete, labeled cell fraction is concentrated to desired volume for magnetic column enrichment and remains in device chamber. The cell labeling and post-label wash conditions are based on the manual protocol provided by the manufacturer for the CliniMACS Plus CD34 system.	
3	This program is suitable for two-step labeling of any desired cell fraction with a primary antibody/labeling agent followed by a secondary-antibody/agent magnetic bead. The program initiates with the assumption that the cell product to be labeled is in the device chamber. The volume to be labeled is set by the user to allow adjustment of cell, antibody and bead concentrations to desired values. The cell fraction to be labeled is first diluted in PBS/EDTA buffer and then is pelleted in a step-wise fashion. Supernatant is removed to bring the labeling volume to the desired value input by the user. The primary antibody/agent with or without blocking agent is then added and the chamber is cooled to 4°C and gently shaken every 30 seconds for a total incubation period of 30 minutes. The secondary antibody/agent magnetic beads are then added, chamber temperature is maintained at 4°C and the chamber is gently shaken every 30 seconds for a second incubation period of 30 minutes. Following incubation the labeled cell fraction is centrifuged and washed to remove non-bound excess antibody/agent/beads. Once washing is complete, labeled cell fraction is concentrated to desired volume for magnetic column enrichment and remains in device chamber. Labeling volumes below the manufacturer's specific 70ml residual volume for the chamber are achieved using a specific centrifugation sequence which pellets the cells low in the chamber, away from the port at the top of the chamber which under normal conditions would siphon out the cell layer. By packing the cells well	TS100

Supplementary Table 1: Complete Description of Custom, Flexible Program Inventory

Program Number	Program Description	Tubing Set
	below the port and maintaining their position under 150rcf, supernatant can be removed through this port to achieve chamber volumes well below 70ml and appropriate for the labeling of small numbers of cells. By this method, the CliniMACS Prodigy can be used to manipulate much smaller numbers of cells than intended, without the need of using excess reagent to maintain the recommended bead:volume labeling ratio.	
4	This program is suitable for magnetic column based selection of any labeled cell fraction. The program initiates with the assumption that the labeled cell fraction to be selected is in the device chamber. A magnetic column and pre-column on the device are primed with PBS/EDTA buffer. The cell fraction to be selected is passed through the pre-column and over the magnetic column with the magnet turned on. Any component of the labeled fraction which does not bind to either the pre-column or the magnetic column (termed “flow-through”) is collected in a bag labeled “Negative Fraction Bag” included in the pre-fabricated tubing set. Following column loading, bound cells are washed, eluted and re-loaded onto the column at slow speed to increase purity of the enriched fraction. Any component of the labeled fraction which is removed during the wash steps is collected in the Waste Bag included in the pre-fabricated tubing set. Finally, the magnet is turned off and cells bound to the column are released and collected into the bag labeled “Target Cell Bag” included in the pre-fabricated tubing set. The final elution volume is approximately 45 mL.	TS100
5	This program is suitable for preparation, magnetic bead labeling and magnetic column enrichment of any cell product which does not require RBC depletion as part of the preparation. Leukapheresis products are one example of such a product. The labeling component of this program is designed for	TS100

Supplementary Table 1: Complete Description of Custom, Flexible Program Inventory

Program Number	Program Description	Tubing Set
	single step labeling with a directly-conjugated immunomagnetic bead. The program initiates with the assumption that the product to be prepared, labeled and enriched is in the Product Application Bag included in the pre-fabricated tubing set. Product preparation includes several washes in PBS/EDTA buffer to remove platelets and other blood product components which can interfere with labeling and enrichment. The washed cell fraction is then diluted in PBS/EDTA buffer and then is pelleted in a step-wise fashion. Supernatant is removed to bring the labeling volume to the desired user value. Immunomagnetic beads with or without blocking agent are then added and the chamber is cooled to 4°C and gently shaken every 30 seconds for a total incubation period of 30 minutes. Following incubation the labeled cell fraction is centrifuged and washed to remove non-bound excess beads. Once washing is complete, labeled cell fraction is concentrated to desired volume for magnetic column enrichment and remains in device chamber. A magnetic column and pre-column on the device are then primed with PBS/EDTA buffer. The cell fraction to be selected is passed through the pre-column and over the magnetic column with the magnet turned on. Any component of the labeled fraction which does not bind to either the pre-column or the magnetic column (termed “flow-through”) is collected in a bag labeled “Negative Fraction Bag” included in the pre-fabricated tubing set. Following column loading, bound cells are washed, eluted and re-loaded onto the column at slow speed to increase purity of the enriched fraction. Any component of the labeled fraction which is removed during the wash steps is collected in the Waste Bag included in the pre-fabricated tubing set. Finally, the magnet is turned off and cells bound to the column are released and collected into the bag labeled “Target Cell Bag” included in the pre-fabricated tubing set. The final elution volume is	

Supplementary Table 1: Complete Description of Custom, Flexible Program Inventory

Program Number	Program Description	Tubing Set
	approximately 45 mL.	
6	This program is suitable for initiating viral vector transduction or media exchange and liquid component addition to any cell product. The program initiates with the assumption that the cell fraction to be transduced/media exchanged is in the Target Cell Bag included in the pre-fabricated tubing set. The user specifies the volume of the final desired cell suspension and the volume of virus vector or other liquid component to be added to the cell suspension. First the cell suspension is transferred from the Target Cell Bag to the device chamber and the Target Cell Bag is rinsed with media specified for media exchange. Bag rinse is also transferred to the chamber. Media exchange is then performed by three cycles of the following: dilution of the cell suspension to the maximum chamber volume with media specified for media exchange, then step-wise centrifugation to pellet cells in suspension, and finally removal of maximum supernatant volume. During the final supernatant removal, the user-defined final cell suspension volume is obtained. Once media exchange is completed, virus vector or additional liquid component is added to the cell suspension in the chamber. As described under program 3, low chamber volumes are achieved by a special centrifugation sequence and supernatant removal from the upper port of the chamber residing at the chamber wall. This volume reduction is important to achieve an economical volume of vector to be used for transduction.	TS730
7	This program is suitable for sequential addition of any two liquid components to a cell suspension. One example of a two-component addition would be the addition of a second volume of virus vector and additional transduction media during a two-hit cell transduction protocol. The program initiates with the assumption that the cell suspension for component addition is in the device chamber. The	TS730

Supplementary Table 1: Complete Description of Custom, Flexible Program Inventory

Program Number	Program Description	Tubing Set
	user specifies the desired volume of each liquid component to add to the cell suspension. The device first clears the tubing path from the components to the chamber to prevent unwanted contamination. The device then sequentially adds the specified volume of each component to the device chamber.	
8	This program is suitable for culture of any cell product in the device chamber. The program initiates with the assumption that the cells to be cultured are already formulated for culture and are present in the device chamber. The program allows the user to define the gas parameters of N_2, CO_2 and O_2, as well as the chamber temperature. The program also gives the user the option to have the cell suspension gently mixed once every 30 minutes during the incubation period. The device heats the chamber to the desired temperature and mixes the appropriate gas formulation for injection into the chamber. Once the appropriate gas formulation is achieved, the gas mix is injected into the chamber. The device continues the incubation at temperature with a complete gas exchange of the chamber every 15 minutes and mixing as specified until the user terminates the program. The cultured cell suspension remains in the device chamber upon program termination.	TS730
9	This program is suitable for harvest and final formulation of any cell product in the chamber of the device. The program initiates with the assumption that the cell suspension to be harvested and formulated is in the device chamber. The user specifies the volume of the initial cell suspension supernatant for transfer to sufficiently remove unwanted media components from the final formulation. First, the cell suspension in the chamber is centrifuged in a step-wise manner to pellet cells. Once pelleted, the specified supernatant volume is removed. After initial supernatant removal, three washes with simultaneous media exchange are accomplished as follows: first, the pelleted cell suspension is	TS730

Supplementary Table 1: Complete Description of Custom, Flexible Program Inventory

Program Number	Program Description	Tubing Set
	diluted to the maximum chamber volume with final formulation media, then cell suspension is centrifuged in a step-wise manner to pellet cells. Once pelleted, a preset supernatant volume is removed. Following the final wash/media exchange, additional supernatant is removed to bring the final cell product formulation to a total volume of 45mL. The formulated cell product is transferred from the device chamber to the Target Cell Bag attached to the device for removal and application-specific use.	

Supplementary Table 2. Cell counts before and after automated processing of healthy human G-CSF mobilized leukapheresis products.

Sample Parameter	Hu HPC-A Donor 1	Hu HPC-A Donor 2	Hu HPC-A Donor 3*
**Initial HPC-A volume (mL)**	223	227	212
**Initial RBC (10^{11})**	0.86	0.50	0.93
**Initial % Granulocytes**	30.7	ND	ND
**Initial WBC (10^{8})**	563.7	455.0	457.0
**Initial CD34^{+} (%)**	0.6	0.9	0.9
**Initial CD34^{+} (10^{6})**	338.2	413.9	393.0
**Enriched CD34^{+} (10^{6})**	141.7	359.7	233.0
**Enriched CD34^{+} Purity (%)**	98.7	96.0	98.1
**CD34^{+} Yield (% of Initial)**	42.0	86.9	58.1

ND: Not determined; value too low for automated blood cell counter to report.

*Due to chronology of product receipt and limited quantities of clinical LV vector available, this product was not transduced.

Supplementary Table 3. Regulatory safety testing results of products manufactured by semi-automated process.

		Measured Result		
**Test**	**Required Result**	HPC-A Donor 1	HPC-A Donor 2	BM Donor 2
Gram Stain	Negative	Negative	Negative	Negative
3 Day Sterility[†]	Negative	Negative	Negative	Negative
14 Day Sterility[†]	Negative	Negative	Negative	Negative
Mycoplasma	Negative	Negative	Negative	Negative
Endotoxin[ε]	≤ 0.5 EU mL^{-1}	≤ 0.5 EU mL^{-1}	≤ 0.5 EU mL^{-1}	≤ 0.5 EU mL^{-1}
Cell Viability by Trypan Blue Dye Exclusion	≥ 70%	82.5%	91.5%	71.0%

HPC-A: leukapheresis; BM: bone marrow; EU: endotoxin units.

[†] Final release sterility testing performed by LABS™ includes bacterial, fungal and yeast testing over 14-day incubation under USP<71> guidelines in controlled cleanrooms.

[ε]Endotoxin levels determined by kinetic turbidimetric method utilizing limulus amebocyte lysate (LAL) reagent.

Supplementary Table 4. Cell counts before and after automated processing to deplete human bone marrow RBCs from healthy adult donors.

Sample	Species	Initial Volume (mL)	Initial RBC (10^{11})	Initial TNC (10^{8})	Initial $CD34^{+}$ (10^{6})	Depleted Volume (mL)	Depleted RBC (10^{10})	Depleted TNC (10^{8})	Depleted $CD34^{+}$ (10^{6})	% RBC Depleted	%Yield $CD34^{+}$
1	Human (Healthy Donor)	163	6.6	33.5	107.8	90	5.6	22.0	61.6	91.5	57.0
2		96	4.9	22.5	15.6	70	ND	14.6	15.0	ND	96.2
3		93	4.8	44.6	52.3	71	ND	70.4	73.3	ND	140.2
4		95	4.6	54.0	84.2	87	2.8	51.0	110.0	93.9	131.4

Supplementary Table 5. Cell counts before and after automated processing of healthy human bone marrow products.

Sample Parameter	Hu BM Donor 1	Hu BM Donor 2	Hu BM Donor 3*
**Initial BM volume (mL)**	96	93	95
**Initial RBC (10^{11})**	4.9	4.7	4.6
**Initial WBC (10^{8})**	22.5	44.6	54.0
**Initial CD34^{+} (%)**	0.7	1.2	1.6
**Initial CD34^{+} (10^{6})**	15.6	52.3	84.2
**Depleted RBC (10^{10})**	ND	ND	2.8
**Depleted WBC (10^{8})**	14.6	70.4ǂ	51.0
**Depleted CD34^{+} (%)**	1.0	1.0	2.2
**Depleted CD34^{+} (10^{6})**	15.0	73.3ǂ	110.0ǂ
**Enriched CD34^{+} (10^{6})**	8.87	21.8	43.4
**Enriched CD34^{+} Purity (%)**	86.1	72.7	95.3
**CD34^{+} Yield (% of Initial)**	56.8	42.0	51.5

ND: Not determined, cells were counted using a Coulter Counter which does not report RBC content.

Values recorded are higher than the initial product. Counts were repeated a total of three times and values were consistent for all three determinations. Reported value is the average of three determinations.

*Due to chronology of product receipt and limited quantities of clinical LV vector available, this product was not transduced.

Supplementary Table 6. Cell counts before and after automated processing of NHP growth factor primed bone marrow.

Sample Parameter	Z13105	Z13083
**Initial BM volume (mL)**	27.0	35.0
**Initial RBC (10^{11})**	1.74	1.92
**Initial WBC (10^{8})**	37.0	73.0
**Initial CD34^{+} (%)**	8.02	4.7
**Initial CD34^{+} (10^{6})**	296.7	343.1
**Depleted RBC (10^{10})**	2.75	2.66
**Depleted WBC (10^{8})**	30.2	60.6
**Depleted CD34^{+} (%)**	7.3	4.7
**Depleted CD34^{+} (10^{6})**	220.5	286.0
**Enriched CD34^{+} (10^{6})**	72.3	30.2
**Enriched CD34^{+} Purity (%)**	98.2	83.6
**CD34^{+} Yield (% of Initial)**	24.4	8.8

Supplementary Table 7. Characteristics of clinical grade anti-HIV LV vector.

TEST	METHOD	RESULTS
**Identity**		
Vector Function	Infection potential and chemoselection of TZM-bl and MAGI-CCR5 indicator cell lines	>2.5-fold inhibition of infection with HIV BaL<br>>2-fold selection after O6BG/BCNU treatment
Vector Insert	Southern Blot Analysis	Vector size consistent with predicted fragment size and one additional band that is smaller than the predicted vector size*
**Potency**		
Physical Titer	P24 ELISA	6.9×10^{7} pg mL^{-1}
Infectious Titer	Serial dilution on HT1080 cells assessed by qPCR	7.7×10^{8} IU mL^{-1}
**Purity**		
Sterility	Aerobic and anaerobic culture for bacteria and fungus	No growth within 14 days
Mycoplasma	Culture and Vero indicator cells	Negative
In vitro viral assay	Assay on MRC-5, Vero and A549 cells	No CPE or hemadsorption
Replication competent lentivirus (RCL) testing	Co-culture of end production cells with C8166 cells with amplification and indicator phases	No evidence of RCL
	Supernatant testing on C8166 cells with amplification and indicator phases	No evidence of RCL
Endotoxin	Limulus amebocyte lysate	>2.4 and <3.6 EU mL^{-1}
Residual Total DNA	Quantitaive PCR	1.91×10^{7} fg μL^{-1}
Residual benzonase	ELISA	<0.195 ng mL^{-1} at 1:1.11 and 1:10 dilutions
Transfer of Residual E1A	Quantitative PCR	Negative
Transfer of Residual SV40	Quantitative PCR	Negative

*Smaller fragment size corresponds with intronic splicing within the UbiC promoter element.[31]

O6BG: O^6-Benzylguanine, BCNU: carmustine / bis-chloroethylnitrosourea
